# Adherence to Screening Tests for Gynaecological and Colorectal Cancer in Patients with Diabetes in Spain: A Population-Based Study (2014–2020)

**DOI:** 10.3390/jcm13113047

**Published:** 2024-05-22

**Authors:** Luyi Zeng-Zhang, Javier de Miguel-Diez, Ana López-de-Andrés, Rodrigo Jiménez-García, Zichen Ji, Olalla Meizoso-Pita, Cristina Sevillano-Collantes, Jose J. Zamorano-León

**Affiliations:** 1 Endocrinology and Nutrition Department, Infanta Leonor University Hospital, Universidad Complutense de Madrid, 28031 Madrid, Spain; luyizeng@ucm.es (L.Z.-Z.); olalla.meizoso@salud.madrid.org (O.M.-P.); cristina.sevillano@salud.madrid.org (C.S.-C.); 2 Respiratory Care Department, Hospital General Universitario Gregorio Marañón, 28007 Madrid, Spain; javier.miguel@salud.madrid.org (J.d.M.-D.); jizich72@gmail.com (Z.J.); 3 Department of Public Health and Maternal & Child Health, Faculty of Medicine, Universidad Complutense de Madrid, 28040 Madrid, Spain; rodrijim@ucm.es (R.J.-G.); josejzam@ucm.es (J.J.Z.-L.)

**Keywords:** diabetes mellitus, screening tests, gynaecological cancer, colorectal cancer

## Abstract

**Objectives**: Both diabetes mellitus (DM) and gynaecological and colorectal cancers are highly prevalent diseases. Furthermore, the presence of DM constitutes a risk factor and poor prognostic indicator for these types of cancer. This study is based on the European Health Interview Surveys in Spain (EHISS) of 2014 and 2020. It aimed to determine the trends in adherence to screening tests for gynaecological cancers (breast and cervical) and colorectal cancer, compare adherence levels between populations with and without diabetes, and identify predictors of adherence in the population with diabetes. **Methods**: An epidemiological case-control study based on the EHISS data of 2014 and 2020 was conducted. The characteristics of participants who underwent screening tests were analysed based on the presence or absence of DM, and predictors of adherence to these preventive activities were identified. **Results**: A total of 1852 participants with reported DM and 1852 controls without DM, adjusted for age and sex, were included. A higher adherence to mammography was observed in women without diabetes compared to those with diabetes, although statistical significance was not reached (72.9% vs. 68.6%, *p* = 0.068). Similarly, higher Pap smear adherence was observed in the population without diabetes in the age group between 60 and 69 years compared to the population with diabetes (54.0% vs. 45.8%, *p* = 0.016). Pap smear adherence among women with diabetes was significantly higher in the EHISS of 2020 (52.0% in 2014 vs. 61.0% in 2020, *p* = 0.010), as was the case for faecal occult blood testing (13.8% in 2014 vs. 33.8% in 2020, *p* < 0.001), but it was not significant for mammography (70.4% in 2014 vs. 66.8% in 2020, *p* = 0.301). Overall, the predictors of adherence to screening tests were older age, history of cancer and higher education level. **Conclusions**: Adherence levels to cancer screening tests were lower in the population with diabetes compared to those without diabetes, although an improvement in Pap smear and faecal occult blood test adherence was observed in 2020 compared to 2014. Understanding predictors is important to improve adherence rates in the population with diabetes.

## 1. Introduction

Diabetes mellitus (DM) is among the most prevalent chronic diseases in the Spanish population, with its prevalence doubling in recent years from 7.6% to 14.8%, positioning Spain as the country with the second-highest prevalence in Europe [1]. It is associated with the presence of multiple comorbidities, including various types of cancer [2]. Specifically, an estimated 1.2- to 1.5-fold increase in the relative risk of developing breast and colorectal cancer (CRC) has been observed in patients with type 2 DM, alongside an increased risk of cervical cancer in those with type 1 DM. Hyperglycaemia, hyperinsulinism and the association with obesity have been postulated as possible pathogenic mechanisms whose role in the development of gynaecological and CRC is widely known [3,4].

In Spain, cancer remains a leading cause of mortality, with a reported incidence of 279,260 new cases in 2023. Of these, 42,721 were CRC, 35,001 were breast cancer and 2326 were cervical cancer [5].

To facilitate early detection of the most prevalent cancers, various screening programmes for breast, cervical and CRC have been progressively implemented in Spain since 1990 [6]. Mammography is used for breast cancer screening in women aged 50 to 69 years, and from 40 years onward for those with risk factors, with screenings scheduled every two years [7]. Cervical cancer screening targets women aged 25 to 64 years through cervical cytology, also known as the Papanicolaou (Pap) smear, conducted every three years. Since 2019, human papillomavirus testing has been incorporated into cervical cancer screening [8,9]. Lastly, CRC screening involves faecal occult blood testing (FOBT) for individuals over 50 years old, recommended biennially [9].

These oncological screening systems have been recognised as effective tools for secondary prevention, enhancing disease prognosis [10,11]. European guidelines stipulate that at least 45% of the target population should undergo CRC screening, and 70% should undergo gynaecological cancer screening, to observe significant beneficial effects at the population level [12,13]. However, previous studies have noted a declining trend in the utilisation of breast and cervical cancer screening programmes among women with DM over the past decades, despite their heightened risk of gynaecological cancers [14].

Therefore, this study aimed to ascertain the temporal trend in adherence to screening tests for gynaecological and CRC based on the European Health Interview Surveys in Spain (EHISS) from 2014 to 2020. It also sought to compare adherence levels between populations with and without diabetes and identify healthcare, sociodemographic and lifestyle variables predictive of adherence to oncological screening programmes among individuals with diabetes.

## 2. Materials and Methods

### 2.1. Design

An observational, retrospective, case-control epidemiological study was conducted using data from the EHISS for 2014 and 2020. In Spain, the EHISS was conducted by the National Statistics Institute under the coordination and supervision of the Ministry of Health. This programme involved personal interviews with a representative sample of the non-institutionalised population aged 15 or older residing in Spain. Participants were selected through multistage probability sampling, with primary-stage units being census sections and secondary-stage units being main family households.

Surveys conducted in 2014 were in-person, whereas those in 2020 were initially in-person until March, then transitioned to telephone interviews due to the impact of the COVID-19 pandemic.

The methodology employed for the EHISS has remained consistent across different editions, enabling the comparison of results obtained in different years, and is detailed by the Spanish National Statistics Institute [15].

### 2.2. Selection of Cases and Controls

All participants reporting a medical diagnosis of DM were classified as cases. Individuals were categorised as cases if they answered affirmatively to the question: “Has a doctor ever diagnosed you with diabetes?” Participants reporting no DM were considered controls. One control was randomly assigned for each case, considering the edition of the EHISS in which they participated, as well as sex, age and place of residence.

### 2.3. Variables

Three dependent variables were selected. The first, mammography as a preventive practice, was assessed by asking participants whether they had undergone a mammogram in the previous two years. The age interval considered suitable for mammography was 40 years or older. The second variable, Pap smear usage, was evaluated by asking participants whether they had undergone a Pap smear in the previous three years. Women aged 18 to 69 years were considered eligible for Pap smear. Lastly, adherence to CRC screening was determined by whether individuals had undergone FOBT in the past two years, with individuals not adhering classified as non-adherents. Both men and women aged 50 to 69 years were considered eligible for FOBT. All variables were derived from questions included in the EHISS, and data were thus self-reported by participants.

The independent variables were:

1. Demographic and clinical characteristics: sex, age, education level, living with a partner, self-rated health and body mass index (BMI).
2. Concomitant diseases: chronic obstructive pulmonary disease (COPD), cardiac ischemia, stroke, cancer, mental illness and high blood pressure (HBP).
3. Risk habits: alcohol consumption, active smoking and sedentary lifestyle.

Detailed descriptions and categories of these variables are presented in Table S1.

### 2.4. Endpoints

The primary endpoints of this study are the following:

1. To establish the temporal trends in adherence to gynecological cancer screening tests and CRC screening tests across the Spanish population using data from the EHISS for the period 2014 to 2020. We will measure adherence as the proportion of the targeted population that completed the recommended screening tests during the aforementioned timeframe.
2. To compare the adherence rates to these screening tests between individuals with and without diabetes. This analysis will involve calculating the percentage of individuals in each group who have undergone the recommended screenings within the prescribed intervals.
3. To identify the key healthcare, sociodemographic and lifestyle predictors of adherence to oncological screening programs among individuals with diabetes. For this, we will perform multivariate logistic regression analyses to determine which factors significantly influence adherence rates, incorporating all independent variables of demographic and clinical characteristics, concomitant diseases and risk habits.

### 2.5. Statistical Analysis

The distribution of the sample of women undergoing breast and cervical cancer screening, as well as men and women undergoing FOBT, was described. All quantitative variables had a normal distribution and were expressed as mean and standard deviation (SD). Qualitative variables were represented as frequency and percentage.

For the comparisons made, quantitative variables were studied using analysis of variance or Student’s *t*-test, and qualitative variables were analysed using the chi-square test. Adherence to the three tests was analysed based on the independent variables of the study, according to the presence or absence of diabetes.

Multivariable analyses were performed using logistic regression; three models were generated based on the absence or presence of diabetes for each oncological screening programme. These models allowed us to evaluate the adjusted change from 2014 to 2020 and predictors of adherence to breast, cervical and CRC screening programmes. The models included variables with a significant association in bivariate analysis or those reported as relevant in the literature.

The level of statistical significance was set at *p* < 0.05 for all comparisons. All statistical analysis was conducted using SPSS version 26.0.

### 2.6. Ethical Considerations

EHISS data are freely accessible and available on the official website of the Ministry of Health of Spain. Because these are anonymised data, according to Spanish legislation, approval by an ethics committee was not required to conduct this study.

## 3. Results

### 3.1. Characteristics of Population with Diabetes According to EHISS Surveys to Assess Adherence to Oncological Screening Programmes

Table 1 depicts the distribution of demographic characteristics, comorbidities, risk habits and performance of screening tests among cases based on the year of EHISS completion. This study included 1852 cases diagnosed with DM and 1852 age-, sex-, EHISS edition- and residence-matched controls. Among the cases, 941 participants (50.8%) were from the 2014 EHISS and 911 (49.2%) belonged to the 2020 EHISS. Of the 2014 EHISS cases, 531 (56.4%) were male and for the 2020 EHISS, this figure was 521 (57.2%), with no significant differences observed. The mean age of cases from the 2014 and 2020 EHISS was also similar at 57.7 years (SD 9.7) and 58.4 years (SD 9.1), respectively (Table 1).

The predominant education level was secondary education, with 707 participants (75.1%) in the 2014 EHISS and 694 (76.2%) in the 2020 EHISS, showing a different distribution of primary and university education between the two surveys. More than half of the cases lived with a partner, specifically, 622 (66.1%) in the 2014 EHISS and 571 (62.7%) in the 2020 EHISS. Most participants perceived their health status as good or very good, specifically, 544 (57.8%) in the 2014 EHISS and 482 (52.9%) in the 2020 EHISS, significantly higher in the 2014 EHISS. The most frequent comorbidity was HBP, with 511 participants (54.3%) in the 2014 EHISS and 485 (53.2%) in the 2020 EHISS. Significant differences were also observed in BMI distribution, with more obesity among individuals with diabetes in 2014 (Table 1).

Regarding the performance of screening tests, no significant differences were observed in the breast cancer programme: 266 women (70.4%) in 2014 and 244 (66.8%) in 2020 reported having undergone mammography in the two years preceding the survey. However, the Pap smear adherence rate among women with diabetes was significantly higher in 2020 (52.0% in 2014 vs. 61.0% in 2020, *p* = 0.010). Conversely, a significant increase was seen in individuals with diabetes reporting having undergone FOBT in the two years before the 2020 survey compared to the 2014 survey (13.8% in 2014 vs. 33.8% in 2020, *p* < 0.001). This increase in FOBT adherence in 2020 was observed in both sexes (Table 1).

### 3.2. Comparison of Mammography Adherence Rates between Women with and without Diabetes according to Sociodemographic, Health and Lifestyle Variables

Table 2 displays the comparison of characteristics of participants who reported undergoing mammography based on the presence or absence of DM. The results revealed higher adherence among women without diabetes, although the difference was not statistically significant. Moreover, greater adherence was observed in women without diabetes who perceived their health status as good or very good, compared to women with diabetes (72.3% vs. 70.3%, *p* = 0.031). In this regard, a higher proportion of adherence to mammography was found in women without diabetes who reported no cardiac ischemia (73.3% vs. 68.3%, *p* = 0.041), cancer (72.2% vs. 66.9%, *p* = 0.035), HBP (71.7% vs. 63.3%, *p* = 0.009), or overweight or obesity (BMI < 25 kg/m^2^; 72.6% vs. 60.6%, *p* = 0.006) compared to women with diabetes. Similarly, women without diabetes with active smoking habits showed greater adherence to mammography compared to women with diabetes (74.5% vs. 62.5%, *p* = 0.026).

Variables such as age, living with a partner and alcohol consumption were associated with mammography adherence in the group of women without diabetes, whereas age, cancer diagnosis and HBP were significantly associated with undergoing mammography in women with diabetes. For the total population, age, living with a partner, cancer diagnosis and HBP were associated with adherence to breast cancer screening.

### 3.3. Comparison of Pap Smear Adherence Rates between Women with and without Diabetes according to Sociodemographic, Health and Lifestyle Variables

Table 3 presents the data comparing the characteristics of patients who reported undergoing Pap smears, depending on the presence or absence of DM. A higher adherence was observed in the population without diabetes in the age group between 60 and 69 years compared to the population with diabetes (54.0% vs. 45.8%, *p* = 0.016). Regarding comorbidities, a higher percentage of women without diabetes undergoing Pap smears reported no COPD (61.8% vs. 56.4%, *p* = 0.041), no cancer (68.4% vs. 49.3%, *p* = 0.031) and no HBP (60.0% vs. 49.6%, *p* = 0.015) compared to the population with diabetes. Conversely, for those reporting mental illness, the highest adherence percentage was observed among women with diabetes (61.2% vs. 56.2%, *p* = 0.005).

Variables such as age, education level, self-rated health, diagnosis of cardiac ischemia, stroke, alcohol consumption, sedentary lifestyle and BMI were associated with Pap smear adherence in the women without diabetes group, whereas variables such as age, education level, diagnosis of HBP, sedentary lifestyle, BMI and survey year were significantly associated with undergoing Pap smear testing in women with diabetes. For the total population, age, education level, living with a partner, self-rated health, diagnosis of cardiac ischemia, history of stroke and HBP, alcohol consumption, active smoking, sedentary lifestyle, BMI and survey year were associated with adherence to cervical cancer screening.

### 3.4. Comparison of Adherence Rates to FOBT between Individuals with and without Diabetes according to Sociodemographic, Health and Lifestyle Variables

Table 4 presents the comparison of characteristics among participants aged over 50 years who reported undergoing FOBT based on the positive or negative diagnosis of DM. Significant differences in adherence between populations with and without diabetes were observed concerning only education level, showing a higher adherence percentage among individuals with diabetes with a lower education level compared to those without diabetes (19.0% vs. 9.6%, *p* = 0.017).

Variables such as age, education level, diagnosis of stroke or cancer, alcohol consumption, active smoking, sedentary lifestyle and survey year were associated with FOBT adherence in the population without diabetes, whereas variables such as living with a partner, sedentary lifestyle and survey year were significantly associated with undergoing FOBT in the population with diabetes. For the total population, age, education level, living with a partner, cancer diagnosis, sedentary lifestyle and survey year were associated with adherence to CRC screening.

### 3.5. Predictors of Adherence to Breast Cancer Screening Programme Based on Diabetes Diagnosis

Table 5 displays the predictors of adherence to mammography among female participants based on the presence or absence of diabetes and in the total study population. In women without diabetes, age over 50 years, living with a partner and alcohol consumption were significantly associated with better adherence to mammography. In women with diabetes, age over 50 years and a history of cancer were associated with higher adherence to this diagnostic test. In the total participant cohort, age over 50 years, living with a partner and cancer diagnosis were associated with better adherence to mammography. Conversely, the presence of DM was associated with lower adherence to this screening procedure.

### 3.6. Predictors of Adherence to Cervical Cancer Screening Programmes Based on Diabetes Diagnosis

Table 6 presents the predictors of Pap smear adherence among female participants based on the presence or absence of diabetes and in both populations. In participants without diabetes, only having a secondary or higher education level was associated with better Pap smear adherence. In participants with diabetes, both having a secondary or higher education level and participating in the 2020 EHISS were associated with higher Pap smear adherence. In the overall female participant cohort, the same associations as in participants with diabetes were observed. However, a sedentary lifestyle was identified as a predictor of poorer adherence to undergoing this screening test. Diabetes was not associated with adherence after multivariable adjustment.

### 3.7. Predictors of Adherence to CRC Screening Programme Based on Diabetes Diagnosis

Table 7 compiles the predictors of adherence to FOBT among participants aged over 50 years based on the presence or absence of diabetes and in both populations. In individuals without diabetes, age over 60 years, secondary or higher education level, a history of cancer and participation in the 2020 EHISS group were predictors of better adherence to FOBT. However, a sedentary lifestyle was identified as a predictor of poorer adherence to this diagnostic test. In individuals with diabetes, only participation in the 2020 EHISS was a predictor of better adherence to FOBT. In the total participant cohort aged over 50 years, age over 60 years, secondary education level, a history of cancer and participation in the 2020 EHISS were associated with better adherence to FOBT. However, a sedentary lifestyle was associated with poorer adherence to undergoing this screening test.

## 4. Discussion

We analysed the temporal trend of adherence rates to breast, cervical and CRC screening programmes among individuals with diabetes residing in Spain, based on data from the EHISS surveys conducted in 2014 and 2020. The results revealed lower adherence to the studied oncological screening programmes among the population with diabetes compared to those without diabetes, with a significant increase in adherence to preventive Pap smear and FOBT tests among patients with diabetes from 2014 to 2020.

The results regarding adherence to preventive cervical cancer screening showed a 9% significant increase from 52.0% in 2014 to 61.0% in 2020 among women with diabetes aged 18 to 69 years. Additionally, adherence to FOBT rose by 20% in 2020 compared to 2014 among the population with diabetes. This increase suggests a shift in trend regarding adherence to cervical and CRC screening programmes compared to previous years. Previous studies have reported stable and lower adherence levels over time among the Spanish population with diabetes for both screening programmes [14,16]. Concerning mammography, the findings did not show significant differences between 2014 and 2020 among women with diabetes. However, notably, our study found a significant decrease in adherence to gynaecological screening programmes among individuals with diabetes compared to those without diabetes. Previous studies have also shown lower adherence to breast and cervical cancer screening programmes among individuals with diabetes compared to those without diabetes [14,17,18,19,20,21]. Similarly, prior studies have identified reduced adherence to FOBT among the population with diabetes compared to those without diabetes [17,18,19]. However, our study did not observe this difference, consistent with another prior study conducted in Spain [16].

A possible reason for the lower adherence to oncological screening programmes among people with diabetes could be that disease control might compete with preventive care, with patients focusing on clinical diabetes control and perceiving long-term disease prevention as less important, even though they may be related to diabetes [22,23]. Different studies have described that physicians and patients tend to prioritise demands and only deal with the most pressing or symptomatic problems, leading to clinical inertia [14,20].

Among the adults with diabetes in our study, several factors associated with higher adherence to the oncological screenings analysed were found in both populations, with and without diabetes. Age was identified as a predictor of mammography in both groups, with and without diabetes. Consistent with other studies, adherence to mammography increased with age up to 69 years [24,25,26]. Additionally, living with a partner was a positive factor for mammography adherence in women without diabetes. This finding coincides with previous studies, suggesting that women living with a partner may be more motivated by their family members to undergo preventive tests [24,25].

Surprisingly, moderate alcohol consumption was associated with higher adherence to breast cancer screening. This result may seem controversial; however, in Spain, moderate alcohol consumption is common among women of higher socioeconomic status, and higher socioeconomic status is associated with greater acceptance of cancer screening tests. Regarding women with diabetes, the only factor in addition to age that seemed to affect adherence to mammography was, interestingly, cancer diagnosis. Previous studies have also established a positive relationship between higher comorbidity and adherence to breast cancer screening in women with diabetes [16].

In previous studies, more mammograms were performed in the population with diabetes [27]. However, in our study, the presence of diabetes was identified as a predictor of poorer adherence to mammography, as previously described in a study conducted in Spain in 2009 [20]. Understanding the factors influencing adherence to mammography in patients with diabetes is important because this population has a higher risk of breast cancer [28] and a higher risk of mortality exists in women with diabetes with breast cancer compared to those without diabetes [29].

As expected, a higher level of education was positively associated with adherence to cervical cancer screening in both populations, with and without diabetes. This finding is consistent with other studies [30]. Higher education level and socioeconomic status have been associated with greater use of preventive services and cancer screening rates, especially in the case of cervical cancer, whose screening is based on an opportunistic system [25]. In the group of women with diabetes, the survey edition was identified as a positive predictor, suggesting that, albeit insufficiently, women with diabetes seemed to progressively adhere to Pap smears.

In both groups, with and without diabetes, education level was a predictor of CRC screening adherence. Among the analysed parameters, age, education level, cancer diagnosis and sedentary lifestyle were predictors of adherence to CRC screening in individuals without diabetes. In line with the programmes previously described, a previous diagnosis of cancer and a sense of greater vulnerability to CRC risk could explain higher adherence in subjects aged 60–69 years [31,32]. Regarding the finding that a higher education level was associated with greater adherence in people without diabetes, other authors have reported similar results [33,34], suggesting that knowing the signs or symptoms of CRC is associated with the use of procedures for this type of cancer and staying current with screening [35,36]. Interestingly, healthy behaviours such as an active lifestyle may reflect individual perceptions of the importance of overall good health, prevention of health risks and perceived risk [37,38]. This reasoning could explain our results regarding the negative association of sedentary behaviour with CRC screening. Finally, in both groups, with and without diabetes, the edition of EHISS was a predictor. The progressive improvement in adherence levels to FOBT over time seems to reflect its gradual implementation since 2000 in Spain, with the programme being introduced at different times in each region [9]. In this sense, our group previously identified that the different periods of CRC screening implementation constituted a crucial factor in improving adherence to CRC screening from 2011 to 2017 in the resident population in Spain [39].

Obesity, a large number of chronic diseases and even mental disorders are highly prevalent in the population with diabetes. Interestingly, previous studies have reported that obesity and mental disorders may contribute to the decreased uptake described in the population with diabetes in the present study. Indeed, patients with obesity may face physician bias, which may in turn lead to poor patient–physician relationships and communication and lower rates of screening [40,41]. Furthermore, patients’ self-perceptions of negative body image may affect preferences for screening; factors such as embarrassment and even unwanted advice to lose weight may be considered barriers that affect participation [42]. Conversely, it has been suggested that own hospital attendance due to diabetes or concomitant chronic diseases may be a potential barrier to preventive care. In this regard, providing guideline-adherent chronic disease care also requires more consultation time than physicians have available per patient [43,44,45,46], which may lead to the prioritisation of diabetes-related care over routine preventive care [45,46,47].

As a reflection of these results, a need exists for educational programmes targeting the population with diabetes and healthcare professionals to raise awareness of the importance of undergoing the described preventive services. We propose that primary care professionals actively engage in querying patients with diabetes about their participation in screening tests and provide reminders to those who have not yet completed such assessments. Additionally, interventions that allow increasing acceptance of screening should be studied and implemented, including electronic reminders and active recruitment strategies, such as follow-up phone calls or a second invitation letter.

With the aim of increasing adherence to oncologic screening programs in the population with diabetes, it is also important to point out that shared care between primary care physicians and diabetes specialists has been associated with better adherence to diabetes-related health services and a higher likelihood of receiving breast and cervical cancer screening compared with care by either practitioner alone [48]. Accordingly, this suggests that integrated diabetes management models may therefore improve attention to other recommended preventive health services by offloading diabetes care from primary care physicians. Supporting diabetes self-management as well as emphasising the importance of preventive care in the diabetic population through training courses or communication media may also better screening participation.

Our study has significant strengths. Firstly, the large sample size provides high power to detect small significant statistical differences. Secondly, it included a control group for a more rigorous study. Thirdly, being a nationally representative survey, the sample is representative of the Spanish population. Fourthly, the methodological rigour of the EHISS ensures the validity of the study and allows for the comparison of these data in future studies with the next editions of the same survey. However, our study also has limitations. The most important is that it did not distinguish between type 1 and type 2 diabetes. The pathophysiology of the two types of diabetes is different, so the result obtained jointly may not be fully applicable when dealing with one of the two specific types. Similarly, the survey does not specify the level of diabetes control, or the treatments employed, factors that could influence both quality of life and adherence to screening tests. The data obtained in the surveys also correspond to information reported by the participants and thus may not fully correspond to their clinical situation due to memory biases or socially conditioned responses, which could influence the results and affect the validity of the study. In addition, although most participants completed the survey in person, a portion of the 2020 survey was conducted by telephone due to the COVID-19 pandemic, and the survey did not distinguish the method of contact with the participant. This fact may lead to certain biases derived from this difference. Due to the large sample size and the high number of comparisons made, the type I error may not be negligible. Therefore, caution must be exercised when interpreting the results of this study. Additionally, despite identifying several predictor factors associated with adherence to screening tests, given that this is an observational study, there is a need for caution in interpreting these associations as causal. Finally, due to the standard methodology used for conducting the surveys, there is a lack of relevant data for analysing adherence to screening tests, such as diet, socioeconomic level and psychological barriers.

## 5. Conclusions

The adherence levels to the oncological screening systems implemented in Spain were significantly lower in the population with diabetes compared to the population without diabetes. However, an improvement in Pap smear and FOBT adherence was observed in the 2020 survey compared to the 2014 survey among the population with diabetes, although the figures were still far from desirable adherence levels. A better understanding of adherence predictors is important to improve the rate of screening tests in patients with diabetes, given their higher risk of developing these types of cancer, their lower adherence to the screening tests and their worse prognosis.

## Figures and Tables

**Table 1:** Distribution according to study variables of people with diabetes diagnosis included in the European Health Interview Surveys for Spain (EHISS) conducted in the years 2014 and 2020.

Variable	Categories	EHISS 2014 (N = 941)		EHISS 2020 (N = 911)		*p*-Value
		n	%	n	%	
Gender	Male	531	56.4	521	57.2	0.741
	Female	410	43.6	390	42.8	
Age (Years old)	Mean (SD)	57.7 (9.7)		58.4 (9.1)		0.143
Age groups (Years old)	18–39	57	6.1	49	5.4	0.204
	40–49	117	12.4	87	9.5	
	50–59	274	29.1	280	30.7	
	60–69	493	52.4	495	54.3	
Education level	No studies/primary	140	14.9	99	10.9	0.009
	Secondary	707	75.1	694	76.2	
	High education	94	10.0	118	13.0	
Living with a partner	Yes	622	66.1	571	62.7	0.105
Self-rated health	Fair/poor/very poor	397	42.2	429	47.1	<0.034
	Very good/good	544	57.8	482	52.9	
COPD	Yes	118	12.5	111	12.2	0.816
Cardiac ischemia	Yes	81	8.6	75	8.2	0.771
Stroke	Yes	37	3.9	33	3.6	0.727
Cancer	Yes	65	6.9	61	6.7	0.857
Mental disease	Yes	216	23.0	174	19.1	0.042
High blood pressure	Yes	511	54.3	485	53.2	0.646
Alcohol consumption	Yes	508	54.0	431	47.3	0.174
Active smoking	Yes	238	25.3	228	25.0	0.896
Sedentary lifestyle	Yes	402	42.7	369	40.5	0.379
Body mass index (kg/m^2^)	<25	185	20.5	210	23.8	0.037
	25–29.9	361	40.1	374	42.4	
	≥30	355	39.4	298	33.8	
Mammography (<2 years)	Yes	266	70.4	244	66.8	0.301
Pap smear (<3 years)	Yes	213	52.0	238	61.0	0.010
FOBT (<2 years) total	Yes	106	13.8	262	33.8	<0.001
FOBT (<2 years) men	Yes	63	14.4	195	35.9	<0.001
FOBT (<2 years) women	Yes	43	13.1	67	28.9	<0.001

**Table 2:** Prevalence of Spain's resident population with and without diabetes who underwent screening for breast according to sociodemographic, clinical and lifestyle features.

		Mammography Last 2 Years						
Variables	Categories	No Diabetes		Diabetes		Both		*p*-Value
		n	%	n	%	n	%	
Gender	Male	-	-	-	-	-	-	-
	Female	542	72.9	510	68.6	1052	70.8	0.068
Age groups (years old) ^A,B,C^	18–39	-	-	-	-	-	-	-
	40–49	43	49.4	31	35.6	74	42.5	0.066
	50–59	185	79.7	174	75.0	359	77.4	0.222
	60–69	314	74.1	305	71.9	619	73.0	0.486
Education level	No studies/primary	45	65.2	87	64.9	132	65.0	0.967
	Secondary	400	73.9	368	69.0	768	71.5	0.076
	High education	97	72.9	55	72.4	152	72.7	0.930
Living with a partner ^A,C^	No	342	76.0	312	70.7	654	73.4	0.426
	Yes	199	68.9	198	65.8	397	67.3	0.076
Self-rated health	Fair/poor/very poor	336	73.4	177	65.8	513	70.6	0.551
	Very good/good	206	72.3	333	70.3	539	71.0	0.031
COPD	No	493	73.5	430	68.9	923	71.3	0.070
	Yes	49	68.1	80	67.2	129	67.5	0.906
Cardiac ischemia	No	534	73.3	477	68.3	1011	70.8	0.041
	Yes	8	57.1	33	73.3	41	69.5	0.251
Stroke	No	538	73.3	495	68.8	1033	71.0	0.056
	Yes	4	44.4	15	65.2	19	59.4	0.282
Cancer ^B,C^	No	495	72.2	453	66.9	948	69.6	0.035
	Yes	47	82.5	57	86.4	104	84.6	0.550
Mental disease	No	421	71.7	345	67.9	766	70.0	0.171
	Yes	121	77.6	165	70.2	286	73.1	0.108
High blood pressure ^B,C^	No	388	71.7	212	63.3	600	68.5	0.009
	Yes	154	76.2	298	73.0	452	74.1	0.396
Alcohol consumption ^A^	No	286	69.8	369	68.1	655	68.8	0.581
	Yes	256	76.9	140	70.4	396	74.4	0.095
Active smoking	No	429	72.5	415	70.2	844	71.3	0.404
	Yes	113	74.8	95	62.5	208	68.6	0.026
Sedentary lifestyle	No	348	73.3	285	71.6	633	72.5	0.585
	Yes	194	72.4	225	65.2	419	68.4	0.058
Body mass index (kg/m^2^)	<25	238	72.6	103	60.6	341	68.5	0.006
	25–29.9	184	76.0	177	71.4	361	73.7	0.241
	≥30	95	73.1	200	71.7	295	72.1	0.770
EHISS	2014	280	74.1	266	70.4	546	72.2	0.146
	2020	262	71.8	244	66.8	506	69.3	0.086

^A^: Significant association for population without diabetes; ^B^: Significant association for population with diabetes; ^C^: Significant association for both. *p*-value represents comparison of frequencies between non-diabetes and diabetes populations.

**Table 3:** Prevalence of Spain’s resident population with and without diabetes who underwent screening for cervix cancer according to sociodemographic, clinical and lifestyle features.

		Pap Smear Last 3 Years						
Variables	Categories	No Diabetes		Diabetes		Both		*p*-Value
		n	%	n	%	n	%	
Gender	Male	-	-	-	-	-	-	-
	Female	489	61.1	451	56.4	940	58.8	0.054
Age groups (years old) ^A,B,C^	18–39	38	66.7	41	71.9	79	69.3	0.542
	40–49	60	69.0	65	74.7	125	71.8	0.399
	50–59	162	69.8	151	65.1	313	67.5	0.276
	60–69	229	54.0	194	45.8	423	49.9	0.016
Education level ^A,B,C^	No studies/primary	20	28.6	54	39.4	74	35.7	0.124
	Secondary	353	61.5	334	58.2	687	59.8	0.253
	High education	116	74.4	63	70.8	179	73.1	0.544
Living with a partner ^C^	No	308	64.2	280	58.8	588	61.5	0.347
	Yes	179	56.6	171	52.9	350	54.8	0.090
Self-rated health ^A,C^	Fair/poor/very poor	324	63.9	177	57.8	501	61.6	0.817
	Very good/good	165	56.3	274	55.5	439	55.8	0.085
COPD	No	446	61.8	380	56.4	826	59.2	0.041
	Yes	43	55.1	71	56.3	114	55.9	0.864
Cardiac ischemia ^A,C^	No	485	61.7	429	56.9	914	59.4	0.055
	Yes	4	28.6	22	47.8	26	43.3	0.203
Stroke ^A,C^	No	487	61.6	441	56.8	928	59.2	0.056
	Yes	2	22.2	10	41.7	12	36.4	0.301
Cancer	No	450	60.6	418	57.0	868	58.8	0.167
	Yes	39	68.4	33	49.3	72	58.1	0.031
Mental disease	No	398	62.4	303	54.3	701	58.6	0.318
	Yes	91	56.2	148	61.2	239	59.2	0.005
High blood pressure ^B,C^	No	366	61.5	244	63.7	610	62.4	0.489
	Yes	123	60.0	207	49.6	330	53.1	0.015
Alcohol consumption ^A,C^	No	252	57.0	317	54.3	569	55.5	0.383
	Yes	237	66.2	133	62.1	370	64.7	0.327
Active smoking ^C^	No	381	60.0	343	54.7	724	57.4	0.057
	Yes	108	65.5	108	62.4	216	63.9	0.562
Sedentary lifestyle ^A,B,C^	No	328	64.2	258	60.3	586	62.4	0.218
	Yes	161	55.7	193	51.9	354	53.6	0.328
Body mass index (kg/m^2^) ^A,B,C^	<25	245	67.1	120	62.5	365	65.5	0.275
	25–29.9	154	60.9	147	55.9	301	58.3	0.252
	≥30	72	51.8	164	55.4	236	54.3	0.481
EHISS ^B,C^	2014	238	58.0	213	52.0	451	55.0	0.079
	2020	251	64.4	238	61.0	489	62.7	0.336

^A^: Significant association for population without diabetes; ^B^: Significant association for population with diabetes; ^C^: Significant association for both. *p*-value represents comparison of frequencies between non-diabetes and diabetes populations.

**Table 4:** Prevalence of Spain’s resident population with and without diabetes who underwent screening for colorectal cancer according to sociodemographic, clinical and lifestyle features.

		FOBT Last 2 Years						
Variables	Categories	No Diabetes		Diabetes		Both		*p*-Value
		n	%	n	%	n	%	
Gender	Male	212	23.9	220	24.8	432	24.4	0.658
	Female	139	21.2	148	22.6	287	21.9	0.548
Age groups (years old) ^A,C^	50–59	103	18.6	121	21.8	224	20.2	0.178
	60–69	248	25.1	247	25.0	495	25.1	0.959
Education level ^A,C^	No studies/primary	13	9.6	41	19.0	54	15.3	0.017
	Secondary	271	23.9	289	25.0	560	24.5	0.524
	High education	67	24.7	38	22.2	105	23.8	0.547
Living with a partner ^B,C^	No	233	24.1	260	25.8	493	25.0	0.914
	Yes	117	20.5	108	20.3	225	20.4	0.376
Self-rated health	Fair/poor/very poor	233	22.6	153	23.4	386	22.9	0.642
	Very good/good	118	23.1	215	24.2	333	23.8	0.710
COPD	No	322	22.7	319	23.7	641	23.2	0.517
	Yes	29	23.8	49	24.9	78	24.5	0.824
Cardiac ischemia	No	332	22.4	333	23.8	665	23.1	0.378
	Yes	19	31.7	35	24.6	54	26.7	0.303
Stroke ^A^	No	344	22.7	350	23.7	694	23.2	0.534
	Yes	7	24.1	18	27.7	25	26.6	0.719
Cancer ^A,C^	No	319	22.1	329	23.1	648	22.6	0.504
	Yes	32	32.7	39	32.5	71	32.6	0.981
Mental disease	No	299	22.5	283	23.4	582	22.9	0.600
	Yes	52	24.2	85	25.5	137	25.0	0.724
High blood pressure	No	234	22.4	150	23.8	384	22.9	0.522
	Yes	117	23.5	218	23.9	335	23.8	0.854
Alcohol consumption	No	125	20.4	182	22.5	307	21.6	0.333
	Yes	226	24.4	186	25.4	412	24.8	0.621
Active smoking ^A,C^	No	281	24.2	282	23.9	563	24.0	0.863
	Yes	69	18.2	86	23.8	155	20.9	0.061
Sedentary lifestyle ^A,B,C^	No	251	24.5	229	25.7	480	25.1	0.540
	Yes	100	19.3	139	21.3	239	20.4	0.396
Body mass index (kg/m^2^)	<25	127	23.6	84	27.8	211	25.1	0.177
	25–29.9	152	23.1	147	23.5	299	23.3	0.847
	≥30	60	20.9	129	23.0	189	22.3	0.489
EHISS ^A,B,C^	2014	100	13.0	106	13.8	206	13.4	0.653
	2020	251	32.4	262	33.8	513	33.1	0.553

^A^: Significant association for population without diabetes; ^B^: Significant association for population with diabetes; ^C^: Significant association for both. *p*-value represents comparison of frequencies between non-diabetes and diabetes populations.

**Table 5:** Predictors of adherence to mammography screening in women participating in the European Health Interview Surveys (2014 and 2020) in Spain, according to the presence of diabetes.

Variables	Categories	No Diabetes OR (95% CI)	Diabetes OR (95% CI)	Both OR (95% CI)
Age groups (years old)	40–49	1	1	1
	50–59	4.85 (2.81–8.38)	5.26 (3.06–9.04)	4.88 (3.33–7.15)
	60–69	3.46 (2.12–5.64)	4.08 (2.45–6.80)	3.56 (2.50–5.06)
Living with a partner	No	1	-	1
	Yes	13.24 (1.33–132.01)	-	15.81 (1.73–144.25)
Cancer	No	-	1	1
	Yes	-	2.88 (1.38–6.01)	2.31 (1.38–3.86)
High blood pressure	No	-	1	1
	Yes	-	NS	NS
Alcohol consumption	No	1	-	-
	Yes	1.54 (1.10–2.18)	-	-
Diabetes	No	-	-	1
	Yes	-	-	0.75 (0.59–0.96)
EHISS	2014	1	1	1
	2020	NS	NS	NS

**Table 6:** Predictors of adherence to Pap smear screening in women participating in the European Health Interview Surveys (2014 and 2020) in Spain, according to the presence of diabetes.

Variables	Categories	No Diabetes OR (95% CI)	Diabetes OR (95% CI)	Both OR (95% CI)
Age groups (years old)	18–39	1	1	1
	40–49	NS	NS	NS
	50–59	NS	NS	NS
	60–69	NS	NS	0.53 (0.34–0.83)
Education level	No studies/primary	1	1	1
	Secondary	3.27 (1.85–5.77)	1.55 (1.03–2.31)	2.00 (1.44–2.77)
	High education	4.99 (2.53–9.84)	2.26 (1.23–4.15)	3.20 (1.95–4.68)
Self-rated health	Fair/poor/very poor	NS	-	NS
	Very good/good	1	-	1
Living with a partner	No	-	1	1
	Yes	-	NS	NS
Cardiac ischemia	No	1	-	1
	Yes	NS	-	NS
Stroke	No	1	-	1
	Yes	NS	-	NS
High blood pressure	No	-	1	1
	Yes	-	NS	NS
Alcohol consumption	No	1	-	1
	Yes	NS	-	NS
Active smoking	No	-	-	1
	Yes	-	-	NS
Sedentary lifestyle	No	1	1	1
	Yes	NS	NS	0.79 (0.66–0.95)
Body mass index (kg/m^2^)	<25	1	1	1
	25–29.9	NS	NS	NS
	≥30	NS	NS	NS
Diabetes	No	-	-	1
	Yes	-	-	NS
EHISS	2014	1	1	1
	2020	NS	1.39 (1.03–1.86)	1.28 (1.04–1.58)

**Table 7:** Predictors of adherence to FBOT screening in women participating in the European Health Interview Surveys (2014 and 2020) in Spain, according to the presence of diabetes.

Variables	Categories	No Diabetes OR (95% CI)	Diabetes OR (95% CI)	Both OR (95% CI)
Gender	Male	1	1	1
	Female	NS	NS	NS
Age groups (years old)	18–39	-	-	-
	40–49	-	-	-
	50–59	1	1	1
	60–69	1.43 (1.09–1.87)	NS	1.31 (1.08–1.58)
Education level	No studies/primary	1	-	1
	Secondary	2.69 (1.47–4.92)	-	1.62 (1.18–2.22)
	High education	2.49 (1.29–4.80)	-	NS
Living with a partner	No	-	1	1
	Yes	-	NS	NS
Stroke	No	1	-	-
	Yes	NS	-	-
Cancer	No	1	-	1
	Yes	1.80 (1.13–2.86)	-	1.74 (1.27–2.38)
Active smoking	No	1	-	1
	Yes	NS	-	NS
Sedentary lifestyle	No	1	1	1
	Yes	0.75 (0.57–0.99)	NS	0.79 (0.66–0.95)
Diabetes	No	-	-	1
	Yes	-	-	NS
EHISS	2014	1	1	1
	2020	3.29 (2.52–4.28)	3.23 (2.50–4.17)	3.24 (2.70–3.90)

## Data Availability

The anonymised EHISS datasets are freely accessible and can be downloaded by anyone on the Ministry of Health’s website. https://www.sanidad.gob.es/estadEstudios/estadisticas/EncuestaEuropea/home.htm (accessed on 8 October 2023). All other relevant data are included in the paper.

## Supplementary Materials

The following supporting information can be downloaded at: https://www.mdpi.com/article/10.3390/jcm13113047/s1, Table S1: Definition of variables according to the questions included in the European Health Interview Surveys in Spain conducted in the years 2014 and 2020.
