# Supplementary material for: Adherence to Screening Tests for Gynaecological and Colorectal Cancer in Patients with Diabetes in Spain: A Population-Based Study (2014–2020)
Source: J Clin Med. 2024 May 22;13(11):3047. doi: 10.3390/jcm13113047 (PMC11172449; doi:10.3390/jcm13113047)
Supplement: Supplementary file 1 [file jcm-13-03047-s001.zip › jcm-2993495-supplementary.pdf]

Table S1. Definition of variables according to the questions included in the European Health Interview Surveys in Spain conducted in years 2014 and 2020.

Questions	Description and answer	Variables name	Categories
Has your doctor told you that you are suffering from diabetes?	1. Yes<br>2. No	Diabetes	1. Case<br>2. Control
Uptake of breast cancer screening. It was determined by asking: 'Have you ever undergone a mammogram?'. Those who answered affirmatively were asked a second question, 'When was the last time you had a mammogram?'.	1. In the last 12 months<br>2. Over 1 year but less than 2 years ago<br>3. Over 2 years but less than 3 years ago<br>4. Over 3 years ago<br><br>Subjects who reported that they had undergone their most recent mammogram within the previous two years were considered as 'uptakers'. The remaining subjects were classified as 'non-uptakers'.	Mamography	1. Yes: Options 1 and 2<br>2. No: Options 3 and 4
Uptake of cervical cancer screening. It was determined by asking:<br>'Have you ever undergone cervical cytology?'.<br>Women who answered affirmatively were asked,<br>'When was the last time you underwent cervical cytology?'.	1. In the last 12 months<br>2. Over 1 year but less than 2 years ago<br>3. Over 2 years but less than 3 years ago<br>4. Over 3 years ago<br><br>Women who had undergone cervical cytology within the last three years were considered as 'uptakers'. The other subjects were classified as 'non-uptakers'.	Pap Smear	1. Yes: Options 1 to 3<br>2. No: Option 4
Uptake of colorectal cancer screening. It was determined by asking:<br><br>'Have you ever undergone a fecal occult blood test?'. Those who answered affirmatively were asked a second question, 'When was the last time you had a fecal occult blood test?	1. In the past 12 months<br>2. Over 1 year but less than 2 years ago<br>3. Over 2 years but less than 3 years ago<br>4. Over 3 years but less than 5 years ago<br>5. Over 5 years ago<br><br>Subjects who reported that they had undergone FOBT within the previous two years were considered 'uptake', and the remaining subjects were classified 'not-uptake'.	FOBT	1. Yes: Options 1 and 2<br>2. No: Options 3 to 5
Which is your gender?	1. Male<br>2. Female	Gender	1. Male<br>2. Female
How old are you?	Age in years	Age groups	1. 18-39<br>2. 40-49

			3. 50-59<br>4. 60-69
What level of education have you completed?	1. Does not know how to read or write<br>2. Incomplete primary education<br>3. Complete primary education<br>4. First stage of Secondary Education, with or without a qualification<br>5. Elementary Spanish Upper Secondary Education<br>6. Upper secondary education<br>7. Intermediate vocational training or equivalent<br>8. Advanced vocational training or equivalent<br>9. University studies or equivalent<br>10. Over university (master, PhD….)	Educational level	1. No studies/Primary: Options 1 to 3<br>2. Secondary: Options 4 to 8<br>3. High education: Options 9 and 10
What is your marital status?	1. Single<br>2. Married<br>3. Widower<br>4. Separated<br>5. Divorced	Living with a partner	1. Yes: Option 2<br>2. No: options 1, 3, 4 and 5
In the past twelve month, how is your perception of your general health status?	1. Very good<br>2. Good<br>3. Fair<br>4. Bad<br>5. Very bad	Self-rated health	1. Very good/good: Options 1 and 2<br>2. Fair/poor/very poor: Options 3 to 5
Has your doctor told you that you are suffering from COPD?	1.Yes<br>2.No	COPD	1. Yes<br>2. No
Has your doctor told you that you are suffering from heart diseases (heart failure or coronary disease?	1.Yes<br>2.No	Cardiac ischemia	1. Yes<br>2. No
Has your doctor told you that you are suffering from stroke?	1.Yes<br>2.No	Stroke	1. Yes<br>2. No
Has your doctor told you that you are suffering from cancers?	1.Yes<br>2.No	Cancer	1. Yes<br>2. No
Has your doctor told you that you are suffering from anxiety or depression?	1.Yes<br>2.No	Mental disease	1. Yes<br>2. No

Has your doctor told you that you are suffering from High blood pressure?	1.Yes<br>2.No	High blood pressure	1. Yes<br>2. No
During the past 12 months, how often have you had alcoholic beverages of any kind (i.e. beer, wine, spirits, distilled and mixed drinks, or other alcoholic beverages)?	1. Daily or almost daily<br>2. 5-6 days per week<br>3. 3-4 days per week<br>4. 1-2 days per week<br>5. 2-3 days in a month<br>6. Once a month<br>7. Less than once a month<br>8. Not in the last 12 months, have I stopped drinking<br>9. Never or just a few sips to taste it throughout life	Alcohol consumption	1. Yes: Options 1 to 6<br>2. No: Option 7 to 9
Could you tell me if you smoke?	1. Yes, I smoke daily<br>2. Yes, I smoke, but not daily<br>3. I don't currently smoke but have smoked before<br>4. I neither smoke nor have I ever smoked regularly	Active smoking	1. Yes: Options 1 and 2<br>2. No: Options 3 and 4
Which of these possibilities best describes how often you do some physical activity in your free time?	1. I don't exercise. I occupy my free time almost completely sedentary.<br>2. I do some occasional physical or sports activity<br>3. I do physical activity several times a month<br>4. I do sports or physical training several times a week	Sedentary lifestyle	1. No: Option 1<br>2. Yes: Option 2 to 4
1. Could you tell me how tall you are, approximately, without shoes?<br>2. Could you tell me your weight, approximately, without shoes and clothes?	Body mass index is calculated with the formulae: Weight in kg/ (Height in meters)^2	Body mass index	1. <25<br>2, 25-29.9<br>3. ≥30

COPD, Chronic obstructive pulmonary disease.
